# Widespread and largely unknown prophage activity, diversity, and function in two genera of wheat phyllosphere bacteria

**DOI:** 10.1038/s41396-023-01547-1

**Published:** 2023-11-02

**Authors:** Peter Erdmann Dougherty, Tue Kjærgaard Nielsen, Leise Riber, Helen Helgå Lading, Laura Milena Forero-Junco, Witold Kot, Jos M. Raaijmakers, Lars Hestbjerg Hansen

**Affiliations:** 1 Department of Plant and Environmental Sciences, University of Copenhagen, Frederiksberg, Denmark; 2 Department of Microbial Ecology, Netherlands Institute of Ecology (NIOO-KNAW), Wageningen, The Netherlands

**Keywords:** Bacteriophages, Bacterial genomics, Next-generation sequencing, Microbial ecology

## Abstract

Environmental bacteria host an enormous number of prophages, but their diversity and natural functions remain largely elusive. Here, we investigate prophage activity and diversity in 63 *Erwinia* and *Pseudomonas* strains isolated from flag leaves of wheat grown in a single field. Introducing and validating Virion Induction Profiling Sequencing (VIP-Seq), we identify and quantify the activity of 120 spontaneously induced prophages, discovering that some phyllosphere bacteria produce more than 10^8^ virions/mL in overnight cultures, with significant induction also observed *in planta*. Sequence analyses and plaque assays reveal *E. aphidicola* prophages contribute a majority of intraspecies genetic diversity and divide their bacterial hosts into antagonistic factions engaged in widespread microbial warfare, revealing the importance of prophage-mediated microdiversity. When comparing spontaneously active prophages with predicted prophages we also find insertion sequences are strongly correlated with non-active prophages. In conclusion, we discover widespread and largely unknown prophage diversity and function in phyllosphere bacteria.

## Introduction

Although it has become an often-cited statement that bacteriophages are the most abundant entities on the planet [1], many do not exist as the free-floating virions we generally envision but instead lie integrated as prophages within the genomes of most bacteria. From this perspective, “most” microbiologists work with phages, albeit indirectly and often unknowingly. Consequently, a better understanding of prophage activity and function may yield deep insights into the behaviour of their bacterial hosts.

Representing the dormant stage of a temperate bacteriophage infection, prophages are phage genomes replicated vertically with their host bacteria. Prophages can remain in this state indefinitely, until either induced to produce phage virions and burst out of the cell, or domesticated by mutations rendering them incapable of induction [2]. Although nearly universal in bacterial taxa, prophages appear to be unevenly distributed. A recent study of 10,370 bacterial and archaeal genomes found predicted prophages in 75% of genomes with an average of 3.24 per genome [3, 4]. In contrast to the strictly predatory nature of virulent (non-integrating) phages, these temperate phages constitute a double-edged sword for their bacterial hosts [5]. The vast majority of temperate phage infections result in immediate viral replication and bacterial death [6], but when integrated, prophages may conditionally increase host fitness through virulence factors [7]. Helpfully, prophages can also provide their hosts with resistance to infection by related phages, although the breadth of this resistance varies [8]. Modelling of a two-microbe system also suggests prophage induction can act as a potent, self-replicating weapon for their hosts [9]. However, in contrast to virulent phages, temperate phages are generally prohibited as biocontrol agents due to the potential transmission of virulence factors [10].

Many possible triggers for prophage induction exist, including DNA damage [11], bacterial toxins [12], and phage-encoded communication systems [13]. Many prophages also exhibit so-called spontaneous induction, where lysogens produce detectable levels of free phage in regular bacterial cultures, although evidence suggests “spontaneous” induction is in response to triggers such as DNA damage observed in a subset of the bacterial community [14–16]. Further complicating matters, prophage induction rates are influenced by the choice of media [17] while indirect effects such as quorum sensing [13, 18] may also impact induction rates in unpredictable fashion. Despite these intriguing dynamics, much is unknown about the role of prophages in microbial ecology, and especially in the phyllosphere. Prophage induction is widely observed in gut bacteria [19], with evidence suggesting they may play important roles in modulating their microbial communities [20]. Despite our growing understanding of their importance, prophages are not generally studied within the field of plant-beneficial synthetic communities (SynComs) [21]. There are however indications that prophages are important players in the phyllosphere; phage depletion has been shown to alter bacterial composition [22], although prophages were not investigated. A recent metagenomics study of the wheat phyllosphere also found 24% of phages were predicted to be temperate, while the most plentiful phage was temperate phage *Hamitonella* virus APSE, which can protect aphids from parasitic wasps [23, 24].

Identifying prophages can be difficult. Although bioinformatics tools such as PHASTER [25] and VIBRANT [26] offer in silico prophage prediction from bacterial genome assemblies, sequence-based identification of prophages has limitations, such as determining whether a prophage is viable, and cannot predict the relative induction rates of prophages or under what conditions they are induced. To quantify induced prophages, traditional plaque assays are extensively used. However, since this requires susceptible hosts, many studies have used culture-free techniques (TEM, epifluorescence microscopy, qPCR) [27–29]. More recently, several tools now identify prophage activity by mapping whole genome shotgun (WGS) reads to bacterial assemblies. PropagAtE [30] and hafeZ [31] both search for regions with high read coverage directly (calculating the prophage/host read coverage), while Prophage Tracer [32] searches for discordant reads to estimate prophage excision rates (although it cannot accurately quantify excision in a host with multiple active prophages). However, the high level of background chromosomal coverage limits the detection limit in WGS data. To solve this, the Tranductomics [33] pipeline sequences only the encapsulated DNA of induced phages, improving the detection limit and enabling investigation of transduction patterns (without phage quantification). Building on the best from these tools, we introduce and validate Virion Induction Profiling Sequencing (VIP-Seq). By quantifying DNA concentrations from encapsulated DNA combined with read mapping and examination of discordant reads, VIP-Seq enables both identification and absolute quantification of all induced prophage titres with high sensitivity.

Applying both VIP-Seq and other techniques to a strain collection isolated from a single environment, we conduct one of the first investigations into prophages in the phyllosphere. From 63 newly sequenced *Erwinia* and *Pseudomonas* strains isolated from the flag leaves of wheat grown in a single field, we discover 120 spontaneously induced prophages from 23 novel genera, quantify their titres, finding many are highly induced in overnight cultures. We further show that extensive prophage induction can also occur *in planta* using wheat seedlings. Investigating the significance of microdiversity that would go undiscovered by traditional metagenomic studies, we find prophages to be primary facilitators of intraspecies diversity and warfare, fragmenting their hosts into non-compatible “phagotypes”. Finally, comparisons with bioinformatic prophage predictions also revealed major discrepancies and suggestions of IS-mediated prophage inactivation. Our results find, for the first time, that prophages are prevalent, active, genetically diverse, and impactful in wheat phyllosphere bacteria.

## Materials and methods

### Isolation of phyllosphere isolates

All bacterial strains were isolated in June 2021 from the flag leaves of four wheat cultivars (Sheriff, Heerup, Rembrandt and Kvium) grown in an experimental field in Høje Taastrup, near Copenhagen, Denmark. Wheat flag leaves were picked, pooled, and either washed or blended prior to dilution plating out on *Pseudomonas* Isolation Agar (NutriSelect Plus, Germany) and incubation at 20 °C. 165 colonies were re-streaked for purification at least three times, and stored at −80 °C in 20% (v/v) glycerol.

### Sequencing and analysis of phyllosphere bacteria

These 165 purified phyllosphere bacterial isolates were sequenced with the long-read Oxford Nanopore Technologies (ONT) platform. The strains were inoculated in LB media (10 g/L NaCl, 10 g/L Tryptone, 5 g/L Yeast Extract) and grown overnight for about 16 h at room temperature (approx. 20 °C) with 225 rpm shaking. DNA was isolated from the isolates using the Genomic Mini AX Bacterial 96-well kit (A&A Biotechnology, Poland). Libraries were built using the Rapid Barcoding 96 kit (SQK-RBK110.96) and sequenced on two PromethION flow cells. All software analyses were run with default parameters unless otherwise indicated.

Basecalling was performed with Guppy (5.1.13 + b292f4d) using the super accuracy model. Reads were assembled using Flye (v2.9) [34], and polished twice with Medaka (v1.5.0) [35] using the ‘r941 prom sup g507’ model. Based on Flye assembly information, 15 of these assemblies appeared to be contaminated with multiple chromosomes or otherwise improperly assembled and were discarded, leaving 150 fully assembled bacterial genomes for further investigation (Supplementary Table S1).

Bacterial taxonomy was determined using GTDB-Tk [36]. Whole-genome distances between all isolates were calculated using FastANI (v1.3.3) [37] nucleotide similarities multiplied with the ratio of aligned fractions. Finally, genomes were annotated with Prokka (v1.14.6) [38] with default settings. Using these annotations, each genome was then rearranged to start at the identified *dnaA* genes. To determine pan- and core-genomes for bacterial species clusters, MMseqs2 (v14.7e284) [39] was used to cluster genes at nucleotide level with 0.9 identity and coverage. Core genes were defined as appearing in at least (N-1)/N genomes in a species cluster. Antiphage defence systems were identified using DefenseFinder [40], and CRISPR spacers with evidence levels 2, 3, or 4 were matched to SIPs using BLASTN (blastn-short, DUST disabled, *e*-value cutoff of 1, gap open and gap extend penalty 10) [41].

### Bioinformatic prediction of prophage elements

Using the bacterial genomes as input, prophages were predicted using Phaster (webserver) and VIBRANT (v1.2.1). Both software split their prophage predictions into three categories; for VIBRANT, categories are “high”, “medium”, and “low” confidence, and for PHASTER “intact”, “questionable”, and “incomplete”. For comparative purposes, PHASTER predictions are hereby referred to as “high”, “medium”, and “low” confidence.

Since our interest was the prophage content of the bacteria, we dereplicated clonal/highly similar bacterial isolates based on their predicted prophage content. First, all individual prophage sequences predicted by VIBRANT were stringently clustered by nucleotide similarity using CD-HIT-EST (v4.7) [42] with a 0.98 similarity cutoff, 0.95 length difference cutoff, and word size 10. Subsequently, the bacterial genomes were clustered if they contained the same combination of predicted prophage clusters. This resulted in 61 bacterial clusters, from which one representative strain was randomly selected from each cluster for further analysis. Two additional strains without VIBRANT-predicted prophages were also included, resulting in a total of 63 strains (45 *Erwinia* strains and 18 *Pseudomonas* strains).

### Identification and quantification of active prophages using Virion Induction Profiling Sequencing (VIP-Seq)

We used VIP-Seq to identify and quantify prophages in supernatants of overnight bacterial cultures.

**Fig. 1:**
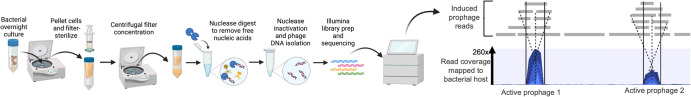
The VIP-Seq workflow used for identification and quantification of active prophages in bacterial cultures. Created with BioRender.com.

In brief, bacterial supernatants were concentrated and DNAse-digested, followed by quantification of the encapsulated DNA and read-mapping back to the bacterial host (Fig. 1). Cultures were inoculated in LB medium and grown overnight (approx. 16 h) at 20 °C with 225 rpm shaking to stationary phase. Cultures were then  centrifuged at 8000 xg for 5 min to pellet cells, and filter-sterilised with 0.22 µm 25 mm cellulose acetate syringe filters (Q-Max, Germany). To increase the concentration of low-titre prophages, 30 mL of the resulting supernatants were concentrated using 100 kDa Amicon filters (Merck Millipore, Ireland), to ≤1 mL. When needed, SM buffer (100 mM NaCl, 10 mM MgSO4, 50 mM Tris-HCl, pH 7.5) was added to adjust the final volume of concentrated supernatants to approximately 1 mL. The supernatants were again filtered with 0.22 µm filters since the Amicon filters are non-sterile.

To remove non-encapsulated nucleic acids, DNase I (25 units/mL) and RNase A (25 µg/mL) (A&A Biotechnology, Poland) were added to the concentrated supernatants and incubated for an hour at 37 °C. Next, DNase and RNAse were deactivated and phage capsids opened by incubating with EDTA (50 µM), SDS (0.1%), and Proteinase K (1 mg/mL) at 55 °C for an hour, followed by Proteinase K deactivation at 70 °C for 10 min.

The raw DNA extractions were then concentrated with Clean & Concentrator-5 (Zymo Research, USA), eluting in 24 µL DNA elution buffer (10 mM Tris, pH 8.5, 0.1 mM EDTA). DNA concentrations were measured on a Qubit 2.0 fluorometer using 5 µL (ThermoFisher, USA) using High Sensitivity dsDNA assays.

To determine the percentage of isolated DNA mapping to active prophage regions, Illumina libraries were built using Ultra II FS DNA Library Prep Kit (New England Biolabs, USA) and sequenced with the Nextseq500 System (Illumina) to generate 150 bp paired-end reads. Reads were trimmed and quality-controlled by running TrimGalore (v0.6.6) [43], and mapped back to their host genomes using CLC Genomics Workbench 2022 (Qiagen, Germany), ignoring non-specific matches. Active prophage regions were found by manually inspecting read coverage, and exact prophage coordinates were identified using discordant reads that map to both ends of the integrated prophage. This allows for manual identification of prophages using very few reads as in Prophage Tracer’s approach [32] but improves detection because there is very little background coverage from chromosomal DNA. In three cases, read coverage was too low to determine the boundaries of the prophage genome directly, and boundaries were instead determined by comparison with highly similar prophages from other strains (Supplementary Table S2). After identifying the active prophages in each bacterial genome, the titre of each induced prophage in each bacterial host was estimated using the formula:

$${induced}\,{prophage}/{mL}=\frac{{m}_{{DNA}}* {N}_{A}}{{M}_{{nt}}* {L}_{{prophage}}}* \frac{{r}_{{pp}}}{{r}_{{tot}}}$$

to obtain induced prophage titres in terms of (induced prophage) genome copies/mL, where ${m}_{{DNA}}$ is the total mass of eluted DNA adjusted per 1 mL overnight bacterial culture, ${N}_{A}$ is Avogadro’s constant, ${M}_{{nt}}$ is the average molar mass of a DNA nucleotide (617.96 g/mol/bp), ${L}_{{prophage}}$ is the length of the induced prophage genome, ${r}_{{pp}}$ is the number of reads mapped to the prophage region, and ${r}_{{tot}}$ is the total number of reads.

For five strains (*E. aphidicola* B01_5, B01_10, W09_2, *P. trivialis* B08_3, and W02_4), the effect of mitomycin C on prophage induction was also investigated. Colonies were inoculated in LB and grown to an OD_600_ of 0.2 after which mitomycin C was added at 1 µg/mL. Following incubation overnight, active prophages were identified, and their activity was quantified as described above.

### Validation of VIP-Seq quantification

To validate VIP-Seq quantification of active prophages, we compared results with those obtained by two other established methods, epifluorescent microscopy (EPI) of virus-like particles (VLPs) stained with SYBR Gold (ThermoFisher, USA), and a traditional plaque assay using susceptible bacterial strains as hosts.

Three phyllosphere strains were chosen for workflow validation; the prophage-inducing strains *E. aphidicola* B01_5 and *E*. sp W01_1, and the *P. trivialis* strain W02_4 presumed not to harbour spontaneously active prophages (although a mitomycin C-induced prophage was found). For comparison, the titre of a T4 phage stock was also estimated using the three methods.

Supernatants from overnight cultures were prepared as before in biological triplicates. A T4 enrichment was obtained by adding 10 µL of a T4 stock (SM buffer) to an exponential-phase *Escherichia coli* MG1655 culture in 100 mL LB and incubated overnight at 37 °C with 225 rpm shaking (also in triplicate). Supernatant was prepared as before.

Active prophage titres from each of the samples were quantified with VIP-Seq. The T4 parallels were also quantified by DNA concentration, although the phage stock was already at high titre and the Amicon concentration step was skipped. After sequencing, T4 reads were assembled using SPAdes [44] 3.13.1, and reads were subsequently mapped to the resulting T4 assembly to quantify the percentage of mapping reads.

EPI microscopy was performed in the following manner. First, DNAse (25 units) and RNAse (2.5 µg) were added to the unconcentrated supernatants and incubated for an hour at 37 °C to remove free nucleic acids. Then, the 10 µL supernatant was added to 990 µL nuclease-free water along with 10 µL 100x SYBR Gold. After vortexing, the samples were incubated at room temperature in the dark for 15 min and then mixed with water to 5 mL. The samples were vacuum filtered using Anodisc 25 0.02 µm (Whatman, UK) filters to trap the VLPs after pre-wetting by running 5 mL water through them. The filters were then transferred to microscopy slides, where a drop of Olympus immersion oil was added to each, followed by a cover glass. The slides were inspected using a Axioplan 2 (Zeiss, Germany) at 1000x, and VLPs were counted manually within a grid.

The samples were also quantified using plaque assays. For T4, *E. coli* MG1655 was used as the host. For the three phyllosphere raw supernatants, the entire collection of 65 strains was screened to find the most efficient host for plaquing (*E. aphidicola* B01_10 for B01_5 supernatant, and *E. aphidicola* B01_5 for W01_1 supernatant). Both supernatants appeared to produce uniform plaques on their respective hosts, indicative of plaquing from a single prophage. To identify which induced prophage was plaquing, three plaques from each supernatant-host pair were scraped, respectively pooled together, and sequenced according to the DPS protocol [45]. The W02_4 supernatant was not observed to plaque on any tested strain.

A dilution series was made for each of the parallels with SM buffer, and 5 µL of each dilution was spotted in triplicate on LB agar plates (100 mm diameter) overlaid with 100 µL of the appropriate overnight culture mixed with 4 mL top agarose (LB medium with 0.4 % agarose, 10 mM MgCl_2_ and CaCl_2_). After overnight incubation at 20 °C (37 °C for the T4-MG1655 plates), plaques were counted to obtain phage titres in terms of plaque-forming units (PFU/mL).

### Bioinformatic analysis of prophages

All identified active prophage genomes were clustered using VIRIDIC [46] (webserver) intergenomic similarity at species (95%) and genus (70%) levels. All prophages were annotated with three different tools; VIGA (v0.11.0) [47], BLAST (v2.12.0), and HH-suite3 (v3.3.0) [48]. With BLASTN, predicted proteins were searched against RefSeq’s viral and bacterial proteins, while HH-suite was used with UniRef30, pdb70, PFAM, scop70, and NCBI_CD databases. Final annotations were chosen in order of priority by BLAST, VIGA, and, HH-suite3. Annotated prophages were aligned, and their synteny visualised at amino acid level using clinker (v0.0.26) [49].

To investigate the similarity between the identified prophages and existing database entries, two BLASTN searches were conducted against the NCBI nucleotide collection (as of January 2023) for each species representative; one against all bacterial sequences, and the other against all virus sequences. Hits were scored according to total BLAST score.

For each experimentally verified active prophage, PHASTER and VIBRANT predictions were compared using three metrics:

$${nucleotide}\,{precision}=\frac{{{\#}}\,{nucleotides}\,{predicted}\,{AND}\,{found}\,{in}\,{active}\,{prophage}}{{{\#}}\,{nucleotides}\,{in}\,{prediction}}$$

$${nucleotide}\,{recall}=\frac{{{\#}}\,{nucleotides}\,{predicted}\,{AND}\,{found}\,{in}\,{active}\,{prophage}}{{{\#}}\,{nucelotides}\,{found}\,{in}\,{active}\,{prophage}}$$

$${active}\,{prophage}\,{recall}=\frac{{Total}\,{{\#}}\,{prophages}\,{predicted}\,{AND}\,{observed}\,{active}}{{Total}\,{{\#}}\,{prophages}\,{observed}\,{active}}$$

For a prophage to be considered “predicted”, we required base recall >0.75, i.e., the prediction covers >75% of the experimentally verified prophage.

### Supernatant host range assay

We evaluated the ability of phyllosphere prophages to infect and kill rival phyllosphere strains. Overnight cultures of all 63 strains were pelleted and syringe-filtered with 0.22 µm filters. Overnight cultures were mixed with 8 mL top agarose and plated on LB agar plates (150 mm). 2 µL of filtered supernatant from each of the 45 *Erwinia* strains was spotted on each *Erwinia* lawn, and likewise for the 18 *Pseudomonas* strains. The plates were incubated overnight at 20 °C and examined for plaques and clearing zones.

To discriminate between productive phage infections (plaques) and clearing zones produced by other means, each clearing zone where plaques were not clearly visible was selected for further experimentation. A dilution series for each of these supernatants was prepared in SM buffer, and 2 µL of these supernatants were again spotted on the relevant hosts. In this manner, each supernatant-host interaction could be classified as plaquing/non-plaquing.

### *In planta* detection of active prophages

In addition to examining prophage induction in vitro, we investigated the production of active prophages *in planta*. *E. aphidicola* strain B01_5, which harbours active prophages, was chosen for the *in planta* trial. Seeds from the winter wheat cultivar Heerup were planted in non-sterile soil and grown in a growth chamber at 22 °C for 12 days with a 16:8 h light:dark cycle. An overnight culture of B01_5 was pelleted at 4,000 x g for 10 min and resuspended in PBS (137 mM NaCl, 2.7 mM KCl, 10 mM Na_2_HPO_4_, 1.8 mM KH_2_PO_4_). This step was repeated once to remove most of the induced prophages in culture. This cell suspension was then inoculated on the first leaf of 12-day-old wheat seedlings (20 seedlings total) using a spray bottle until the leaf was completely covered. To control for the presence of already-induced prophages in the PBS-buffered cell suspension, this cell suspension was pelleted again and the supernatant syringe-filtered with a 0.22 µm filter to remove bacterial cells. This cell-free supernatant was then inoculated on the first leaf of 12-day-old wheat seedlings (20 seedlings total) in the same manner as before.

After inoculation onto the wheat leaves, the populations of B01_5 and active prophage Glittertind_A were monitored over time in the following manner. For each time point (0, 1, 2, 3, and 5 days post inoculation), the first leaf of four seedlings inoculated with the cell suspension, and four seedlings inoculated with the control supernatant were harvested and placed in falcon tubes with 3 mL SM buffer. The tubes were shaken horizontally at 350 rpm for 30 min, and subsequently vortexed at max speed for 10 s. Leaf washes were diluted in SM buffer and plated on PSA for colony counts, as well as syringe-filtered with 0.22 µm filters for plaque assays with the susceptible host *E. aphidicola* B01_10. Up to 500 µL leaf wash was plated, equivalent to a detection limit of 6 CFU/leaf or PFU/leaf (1.5/leaf for the mean of four replicates). All plates were incubated at 20 °C, with plaques counted after overnight incubation. The colonies of strain B01_5 grown on the PSA plates were counted after two days incubation.

### Graphics software

Graphics were created with BioRender.com, clinker (v0.0.27) [49], and the R programming language (v4.2.2) [50] with packages ggplot2 (v3.4.1) [51], phyloseq (v1.42.0) [52], ggtree (v3.6.2) [53], ggtreeExtra (v1.8.1) [54], ggridges (v0.5.4) [55], and ggsankey (v0.0.99999) [56].

## Results

### Collection and sequencing of phyllosphere bacterial isolates

150 *Erwinia* and *Pseudomonas* strains were isolated in June 2021 from the flag leaves of four wheat cultivars (Sheriff, Heerup, Rembrandt, and Kvium) grown in an experimental field in Høje Taastrup, near Copenhagen, Denmark. These isolates were sequenced, and their chromosomes assembled into single contigs. Bacterial taxonomy was assigned at the species level for all isolates (Supplementary Table S1), and the prophage content of each genome was predicted bioinformatically using PHASTER [25] and VIBRANT [26]. To dereplicate highly similar bacterial strains and retain all predicted prophages, VIBRANT prophage predictions were used to dereplicate hosts by predicted prophage content, clustered by nucleotide similarity, resulting in 61 bacterial clusters. One bacterial isolate was randomly chosen from each cluster for further analysis. Two isolates with no predicted prophages were also included, resulting in a total of 63 strains in five species-level clusters: *Erwinia aphidicola* (40 strains), *Erwinia billingiae* (1 strain), *Pseudomonas poae* (12 strains), *Pseudomonas trivialis* (6 strains), and a presumed novel species cluster (*Erwinia* sp*.)* of four strains related to *E. billingiae* (fastANI [37] 0.84, alignment fraction 0.71).

### *Erwinia* and *Pseudomonas* strains from the phyllosphere harbour diverse prophages spontaneously induced at high titres

The 63 selected bacterial isolates were separately grown overnight in liquid lysogeny broth (LB). Using VIP-Seq, we investigated the spontaneously induced prophages (SIPs) in each culture. In total, exact chromosomal locations of 120 SIPs were identified, and their titres quantified (Supplementary Table S2). In two additional cases, read coverage indicated a probable prophage region (*E. aphidicola* B03_6 and *P. poae* B05_3) but it was not possible to determine the exact boundaries of their genomes and they were therefore dropped from analyses.

After SIP identification, VIRIDIC [46] clustered the 120 SIPs into 28 species-level (95% genomic similarity) and 23 genus-level (70% genomic similarity) clusters. Each species-level cluster was named after a Norwegian mountain, with individual prophages within the cluster named with alphabetical suffices (for example phages Galdhoepiggen_A, Galdhoepiggen_B) in the species cluster Galdhoepiggen.

**Fig. 2:**
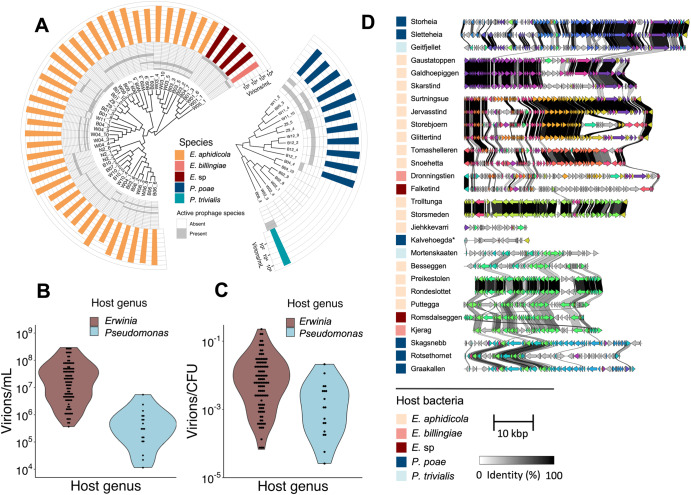
Overview of the bacterial strains and their SIP content. **A** Cladograms of 45 *Erwinia* and 18 *Pseudomonas* strains based on whole-genome similarity (UPGMA clustering). Also shown are the active prophages in each strain at species-level clusters. Finally, the aggregated virion titre in overnight culture for each strain is shown in a bar-plot. **B** Violin plot of estimated virions/mL for all 120 *Erwinia* and *Pseudomonas* active prophages. **C** Violin plot of estimated virions/CFU for all 120 *Erwinia* and *Pseudomonas* active prophages. **D** Representatives from each of the 28 identified prophage species-level clusters labelled with the name of each cluster. The Kalvehoegda cluster is marked “*”, as annotation shows this region is likely a phage satellite. Next to this, clinker figures showing amino acid alignment in coding regions between the species representatives.

Many of the SIPs were highly induced in overnight cultures, with titres up to 3*10^8^ virions/mL (Fig. 2A-B). In some strains, virion titres even rivalled colony-forming units (CFU) counts; in *E. aphidicola* strain Z9_1, the aggregated virion/CFU ratio was 0.32 (Fig. 2C).

There were significant differences between the SIP content of the *Erwinia* and *Pseudomonas* strains (Fig. 2 A-C). In median, the bacterial strains harboured two SIPs, while the *E. aphidicola* strain Z9_3 harboured the most (four). In contrast, six *Pseudomonas* strains appeared to lack SIPs in overnight cultures. *Erwinia* strains also generally had higher aggregated virion titres (median (IQR) 6.5*10^7^ (2.4 – 12)*10^7^ virions/mL) compared to *Pseudomonas* (median (IQR) 5.0*10^5^ (2.9 – 12)*10^5^ virions/mL). Although these differences are reduced after adjusting for CFU concentration (Fig. 2C), they remain significant (*p* = 8.9*10^-4^, Kruskal-Wallis rank sum test).

Examining the SIP genomes reveals many shared genomic segments interspersed with non-aligned regions (Fig. 2D). There was tremendous diversity in the SIPs, with genome lengths ranging from 16-57 kbp. A BLASTN search against bacterial and virus sequences in the NCBI nucleotide database were conducted for each of the prophage species representatives. No high-scoring (query cover * percent identity > 0.7) viral hits were found, indicating each of the 23 genus and 28 species clusters may represent a novel phage genera and species. Meanwhile,16 kbp SIP Kalvehoegda_A (host bacteria *P. poae* B04_4) had no BLASTN hits to virus sequences in the NCBI database. Annotation revealed ash-family proteins and no structural genes, suggestive of a phage satellite dependent upon a helper prophage [57]. No other SIPs could be confidently identified, although both PHASTER and VIBRANT predicted additional prophages in the bacterial genome.

This diverse prophage content strongly contributes to strain-level diversity. To quantify how much intra-species diversity is due to these SIPs, we found the pan-genome of the 40 *E. aphidicola* strains (4771 genes) was divided into a core genome (3896 genes) and an accessory genome (875 genes). Of these accessory genes, 494 were found in SIP regions, representing 56% of gene diversity in the 40 *E. aphidicola* strains. We also found evidence of transduction, further underlining their importance as agents of gene transfer. Using read mapping to investigate transduction patterns as in the Transductomics pipeline [33], we found many of the detected prophages had heightened read coverage in adjacent regions. All eight Storsmeden phages (hosts listed in Supplementary Table S2) exhibited read coverage indicative of lateral transduction of an almost 200 kbp adjacent region, where bacterial chromosomal DNA is packaged in successive capsid headfuls [33, 58–60]. To a lesser extent, members of the closely related Trolltunga species cluster also exhibited lateral transduction in the same region (Supplementary figure S3).

### Mitomycin C treatment reveals additional inducible prophages

Attempting to discover viable prophages not spontaneously induced in overnight cultures, we incubated five strains (*E. aphidicola* B01.5, W09.2, B01.10, *P. trivialis* B08.3, and W02.4*)* with mitomycin C, a DNA-damaging induction trigger for many prophages. W02_4 was previously considered not to harbour active prophages based on both VIP-Seq data from untreated overnight cultures and VIBRANT prophage predictions.

Except for *P. trivialis* prophage Geitfjellet_A, the mitomycin C treatment increased the titres of previously identified SIPs. In addition to the previously established SIPs, novel 21 kbp putative prophages were induced at roughly 1.5*10^6^ virions/mL in both B08_3 and W02_4. Although VIBRANT did not predict these two regions as prophages, PHASTER did predict active prophages in these regions. Precise boundaries for these prophages could not be found with VIP-Seq. In B08_3, this new region was induced at a much higher level than the two known SIPs Geitfjellet_A and Mortenskaaten_A; in fact, the titre of Geitfjellet_A was reduced 10-fold relative to the untreated overnight culture (Supplementary Table S2).

### Validation of VIP-Seq

To validate the VIP-Seq quantification of induced prophages, we compared our protocol with both epifluorescence microscopy (EPI) counts of stained VLPs [61] and PFU counts on susceptible hosts for untreated overnight cultures of B01_5, W01_1, W02_4, and virulent phage T4 (Supplementary Table S4). No SIPs had been identified in untreated W02_4 culture, although a mitomycin C-inducible prophage had been found.

**Fig. 3:**
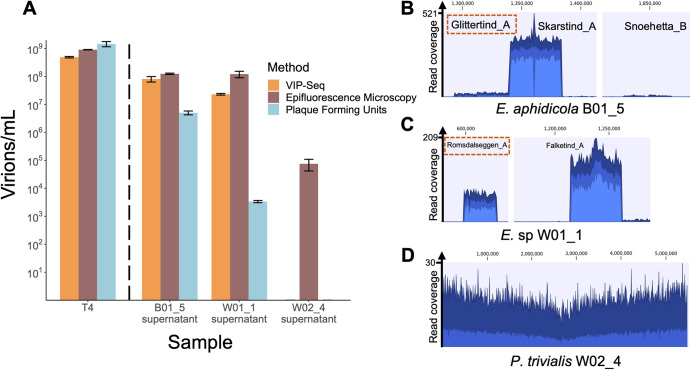
Comparison of VIP-Seq quantification with plaque- and epifluorescent microscopy counts. **A** Comparison of phage titres for *E. coli* virulent phage T4, *E. aphidicola* strain B01_5 SIPs, *E*. sp strain W01_1 SIPs, and *P. trivialis* strain W02_4 SIPs. Prophage titres were measured using VIP-Seq, epifluorescence microscopy, and plaque counts using susceptible strains B01.10 (for B01.5 SIPs), and B01.5 (for W01.1 SIPs). W02_4 supernatant did not plaque on any tested strains. For VIP-Seq, supernatant from B01_5, W01_1, and W02_4 was concentrated using Amicon filters prior to DNA extraction, unlike the higher-titre T4 stock. Each error bar represents the standard deviation of three technical replicates. **B**−**D** Read coverage plots for VIP-Seq libraries built on untreated overnight cultures for B01_5, W01_1, and W02_4 respectively. Prophage regions were enlarged and collated for B01_5 and W01_1 (preserving relative scale), while the whole genome is shown in W02_4. Prophages Glittertind_A and Romsdalseggen_A are boxed in red, indicating they are the plaquing SIPs enumerated in (**A**).

Except for the W02_4 supernatant, VIP-Seq titres agreed well with EPI counts, measuring 54%, 65%, and 19% of EPI counts for T4, B01_5, and W01_1 respectively (Fig. 3). In contrast, PFU was more variable; while plaque counts exceeded EPI counts for T4, the PFU/EPI ratio for B01_5 and W01_1 was 4*10^-2^ and 2.8*10^-5^ respectively. Since both these strains contain multiple SIPs that might form PFUs, we sequenced plaques on both indicator strains to determine which SIP was plaquing (Fig. 3B, C). After adjusting for the relative titres of the plaquing SIPs in the overnight cultures, the adjusted PFU/EPI ratios were 1.05 and 1.3*10^-4^ respectively, demonstrating that PFU can only conditionally be used to quantify induced prophage titres.

In the W02_4 supernatant, VIP-Seq and plaque assays were negative while the EPI count was 2.5*10^4^ VLP/mL (Fig. 3A, D). As LB media contains fluorescent proteins [62], these may not represent true phages. However, W02_4 was shown to contain a mitomycin C-inducible prophage which could also be spontaneously induced at levels undetectable to VIP-Seq.

Performing EPI counts of supernatants before and after 30x Amicon concentration, we found that the concentration step on average resulted in 47% adjusted EPI count loss (Supplementary Table S4). From this, we estimated the detection threshold of VIP-Seq quantification with the utilised parameters (30 mL supernatant, 24 µL eluted DNA, 5 µL for Qubit measurement, 41 kbp average SIP genome). Given the Qubit detection limit of 0.1 ng DNA, this implies a virion detection limit of 7.2*10^5^ virions/mL (well above the W02_4 EPI count of 2.5*10^4^ VLP/mL). Although VIP-Seq cannot measure induction rates, this roughly corresponds to an induction rate of around 2*10^-6^ (using the average CFU/mL of 2.9*10^9^ and λ phage burst size of ~150 [63]. However, detection is also dependent on the relative coverage between multiple active prophages in the same genome, and very-low-titre induced prophages may be detectable if the host also harbours a higher-titre prophage producing more DNA; for example, SIP Storheia_B had a titre of 1.1*10^4^ virions/mL.

### *Erwinia* host prophages capable of bacterial warfare

Having shown SIPs to be widespread in the 63 phyllosphere isolates, we investigated the potential ecological significance of this induction by conducting a host range assay for the supernatant of each strain against all strains of the same genus (Supplementary Table S5).

**Fig. 4:**
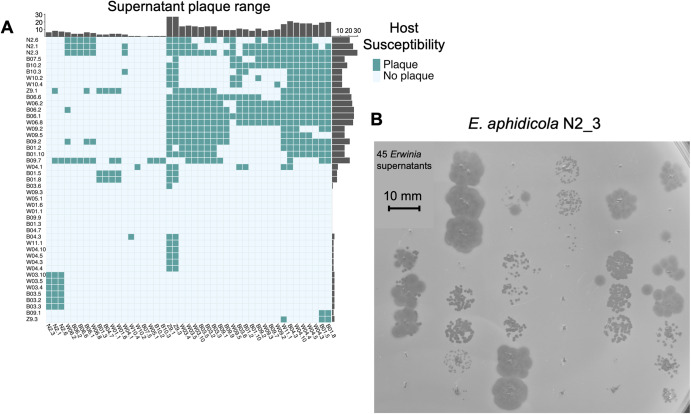
*Erwinia* supernatant plaque assay. **A** Heatmap showing results of the 45 × 45 *Erwinia* supernatant plaque assay, with supernatant host range is shown on the vertical, while host susceptibilities are shown on the horizontal. Barplots displaying cumulative host range/susceptibility show adjacent to respective axes. **B** Plaque assay of the 45 *Erwinia* supernatants upon a lawn of *E. aphidicola* N2.3, demonstrating widespread susceptibility and a variety of plaque morphologies. Image cropped for clarity.

While the SIPs in many *Erwinia* supernatants demonstrated broad host ranges upon their rival strains isolated from the same environment (Fig. 4A), the *Pseudomonas* supernatants failed to produce a single visible plaque, although some turbid clearing zones were observed. There was significant variation in host ranges, with *Erwinia* supernatants plaquing on between 0 (*E. aphidicola* B07.5) and 27 (*E. aphidicola* Z9.1 and Z9.3) rival strains (Fig. 4B). Prophage susceptibility similarly varied, from 0 (multiple strains) to 30 (*E. aphidicola* N2.3). There were also at least two examples of broad range infections, with both *E. billingiae* W05.1 and novel *E*. sp strains plaquing on *E. aphidicola* strains. Some supernatants also displayed multiple plaque morphologies on the same host (for example the left-most three spots in Fig. 4B corresponding to supernatants from W11.1, Z9.1, and Z9.3 containing 2, 3, and 4 identified SIPs respectively), indicating multiple SIPs are probably plaquing.

To investigate these plaquing patterns, we ran DefenseFinder [40] on the 63 strains, identifying 412 phage defence systems divided in 24 types (Supplementary Table S6). The median number of systems in *Erwinia* strains was eight compared to only four in *Pseudomonas* strains. Identifiable defence systems were found on 6/28 of the SIP species clusters. CRISPR systems were also found in all 40 *E. aphidicola* strains. Spacers against the same six SIP species clusters (Glittertind, Surtningssue, Puttegga, Jervasstind, Storebjoern, and Tomashelleren) were found in all but three *E. aphidicola* strains, with additional spacers against the Snoehetta phages were found in *E. aphidicola* B01_2 and *E. aphidicola* B01_10. Auto-immune spacers targeting SIPs in the same genome were also present in 30/40 of the *E. aphidicola* genomes.

To investigate further the defensive properties of all VIP-Seq identified SIPs, we clustered the *Erwinia* strains into 15 “phagotypes” determined by their combinations of SIPs clustered at genus level (70% identity). Of the supernatant interactions between members of the same phagotype (or subset phagotypes), 1/223 (0.45%) were plaquing compared to 448/2025 (22%) of all interactions. This protection was very broad; even when phagotypes were determined using SIPs clustered at 50% nucleotide identity, only 4/364 (1.1%) of relevant interactions were plaquing (Supplementary Table S5). The distinct, antagonistic phagotypes generated upon prophage infection thus appear to hold across evolutionarily diverged prophages, suggestive of a role in the evolutionary divergence of their bacterial hosts as well.

### High levels of prophage induction observed *in planta*

As many *Erwinia* strains produced high levels of SIPs in vitro, we investigated whether this could also occur *in planta*. Using the SIP-producing strain *E. aphidicola* B01_5, we inoculated the first leaf of 12-day-old wheat seedlings with washed overnight cultures of B01_5. As a control, the cell-free supernatant of the washed culture was inoculated on separate seedlings.

**Fig. 5:**
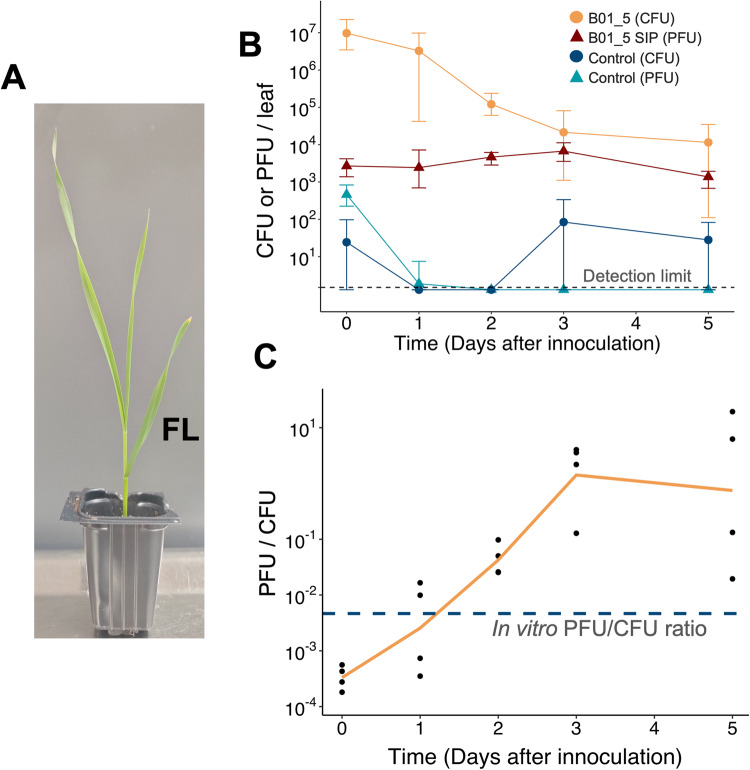
*In planta* SIP production from *E. aphidicola* strain. **A** 12-day-old wheat seedling with the first leaf labelled FL. **B** CFU and PFU of two treatments inoculated on the first leaf of 12-day-old Heerup seedlings. The first treatment (B01_5) is *E. aphidicola* strain B01_5 washed twice in PBS buffer, while the second treatment (control) is the cell-free supernatant of washed B01_5. Leaves were washed in SM buffer, and colonies counted on Pseudomonas selection agar, while plaques were counted using soft-agar overlays with susceptible strain B01_10 as host. Each data point is the mean of four biological replicates, with error bars representing max/min values. The detection limit for the mean of four biological replicates is 1.5 PFU or CFU / leaf (denoted by black dotted line), with datapoints below this artificially set at 1.3 for clarity. **C** The ratio PFU/CFU for the B01_5 cell treatment, along with the recorded in vitro PFU/CFU ratio denoted by the blue dotted line. Orange line connects the mean of each data point, while all data points are shown as black dots.

Next, CFU and PFU (utilising that B01_5 SIP Glittertind_A plaques on B01.10) from both treatment and control were monitored over five days (Fig. 5, Supplementary Table S7). While the PFU of the cell-free control fell below the detection limit by day two, the PFU of the B01_5 treatment stayed relatively stable throughout all five days (Fig. 5B). In fact, a statistically significant increase in PFU was even observed between day 0 and 3 (*p* = 0.04, Kruskal-Wallis rank sum test) before falling by day five. This relative stability in PFU contrasted with the CFU count of the B01_5 count, which fell almost three logs between days zero and five. As a result of these contrasting trends, the PFU/CFU ratio of the B01_5 treatment varied significantly over the course of the time series. Although the PFU/CFU starts well below the in vitro overnight culture PFU/CFU ratio (4.7*10^-3^), by day three it had climbed to >500x the in vitro ratio (Fig. 5C), demonstrating very high induction rates *in planta*.

### Comparing active prophages to bioinformatic predictions suggests possible IS-mediated prophage inactivation

We then compared the 120 identified SIPs to PHASTER and VIBRANT-predicted bioinformatic predictions (Supplementary Table S8).

**Fig. 6:**
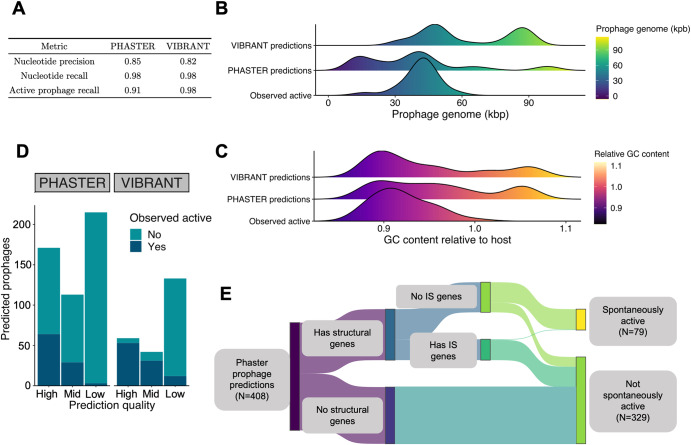
Bioinformatical analysis of SIPs. **A** Performance of PHASTER and VIBRANT prophage predictions relative to the 120 SIPs discovered using VIP-Seq. Performance metrics detailed in Section 2.6. **B** Distribution of prophage region size for high-quality prophage predictions compared to that of all SIPs. **C** Same as (**B**), but with prophage region GC content relative to that of the host bacterial strain. **D** Bar plot showing the number of prophage predictions observed to be spontaneously active for each of PHASTER and VIBRANT’s confidence predictions (labels masked “High”, “Mid”, and “Low” in order of prediction confidence). **E** Sankey diagram illustrating the composition of PHASTER prophage predictions from the 40 *E. aphidicola* genomes.

Checking whether the tools correctly predicted the VIP-Seq-identified SIPs, we found VIBRANT predicted 117/120 (0.98) and PHASTER predicting 109/120 (0.91) (Fig. 6A). The putative phage satellite Kalvehoegda_A was predicted by neither PHASTER nor VIBRANT. Both tools also had relatively low nucleotide precision as they often flanked the VIP-Seq verified prophages with extraneous host DNA. These differences were apparent even when only considering high-confidence prophage predictions, as the distribution of genome size and relative GC content are different from that of experimentally active prophages (Fig. 6B, C). Especially prominent are the very long prophage genome lengths from VIBRANT, partly due to the merging of several pairs of prophages situated closely together (i.e., B01.5 prophages Glittertind_A and Skarstind_A, Fig. 3B) as single prophage predictions.

Beyond predicting >90% of the SIPs, PHASTER and VIBRANT also predicted many prophages not observed as SIPs (Fig. 6D). To explore whether these predictions may constitute viable prophages, we focused on the largest subset of predictions; PHASTER-predicted prophages of the 40 *E. aphidicola* genomes. These 408 prophage predictions were annotated as before, followed by manual curation to identify all structural phage genes (i.e., tail proteins, capsid proteins). Next, all putative insertion sequence (IS) transposases were annotated by running Prokka and flagging every match to the ISfinder database [64]. Finally, each prediction was labelled spontaneously active/inactive based on VIP-Seq data.

Surprisingly, half (53%) of the PHASTER predictions completely lack structural gene annotations and are thus unlikely to be viable prophages; indeed, none of these predictions were SIPs (Fig. 6E, Supplementary Table S9). Furthermore, only 2/79 (2.5%) predictions with both structural and IS annotations were SIPs. Inspection of these two outliers revealed that the IS elements were outside the VIP-Seq verified prophage regions and were merely artefacts of inaccurate PHASTER predictions. Since transposable phages (such as bacteriophage Mu [65]) will contain transposases that may be annotated as IS elements, we also ran BLASTN on these 79 predictions against a database of 18,449 transposable phages [66], but no hits exceeded 10% query alignment.

In contrast to the inactivity of predicted prophages with IS elements, 77/114 (68%) of PHASTER predictions with structural genes and no IS annotations were SIPs. Intriguingly, these results suggest that while structural genes are a necessary condition of prophage viability, IS elements may be strong indicators of prophage inactivation/domestication. Assuming these 114 PHASTER predictions represent the pool of potentially viable prophages in the *E. aphidicola* strains, 77/114 were detected as SIPs using VIP-Seq. However, this is not a conclusive result as we have only identified spontaneously induced prophages, and some predicted prophages with IS elements may be induced non-spontaneously, or induced at rates undetectable to VIP-Seq.

## Discussion

Too influential to be overlooked, the prophages harboured within most bacteria expertly manipulate their hosts, and investigating their dynamics will help us better understand interactions within microbial communities. In this study, we isolated and sequenced a strain collection from wheat flag leaves grown in a single field and investigated the activity of their prophages. We also introduced and validated VIP-Seq, a novel method to precisely identify and quantify spontaneously induced prophages in bacterial isolates, discovering plentiful and active prophages engaged in intraspecies bacterial warfare and diversification.

Many phyllosphere prophages from the investigated strains were spontaneously highly induced, with titres up to 3*10^8^ virions/mL. This is the second-highest spontaneous induction titre ever recorded (a *Salmonella* prophage was recorded at 10^9^ PFU/mL) [16], although methodologies differ. We also demonstrate very high rates of prophage induction can occur *in planta*, vastly exceeding the PFU/CFU ratio observed in vitro. Some prophage induction may also have occurred during the 30 min wash step, but this cannot explain the large increase in the PFU/CFU ratio as all samples were subjected to the same treatment. Although these extreme rates of induction may also be an artefact of stress provoked by inoculation at unnaturally high bacterial titres, they are in line with previous work demonstrating phyllosphere transduction in a *P. aeruginosa* lysogen [67]. As stress will also occur in natural settings, these results certainly show prophage induction can occur *in planta*.

This prophage induction is of significant ecological importance, as we discovered prophage-mediated bacterial warfare exhibited by widespread cross-plaquing between rival *Erwinia* strains. Spontaneously active prophages provided their hosts with broad resistance to rival phages, with additional anti-phage defence systems also present. The *E. aphidicola* genomes all had I-F CRISPR-Cas systems with spacers targeting rival SIPs, although most also had auto-immune spacers targeting their own SIPs, previously shown to be a common feature of type I-F systems [41]. It was however surprising to find such extensive prophage-mediated bacterial antagonism in strains isolated from leaves grown in the same field. This type of dynamic is not caught by traditional metagenomics studies and suggests prophage-mediated bacterial microdiversity may be more impactful than previously thought. Indeed, prophages contributed most of the genetic diversity in *E. aphidicola* isolates and appeared to engage in transduction, potentially enabling horizontal transfer of host genes. The lack of genetic diversity outside prophage regions itself likely aided the broad host range of many Erwinia prophages. An intriguing hypothesis arises from all this, wherein *Erwinia* prophages fragment their phyllosphere hosts into antagonistic “phagotypes” sent on divergent evolutionary trajectories. In contrast however, the *Pseudomonas* strains harboured fewer prophages with lower spontaneous induction rates, fewer predicted anti-phage defence systems, and no observed supernatant plaquing. Although some of this may be an artefact of a smaller sample size, phylogenetic differences in (predicted) prophage content is known to occur [3], and it seems possible that temperate phages play a smaller role in *Pseudomonas* than in *Erwinia*.

We also found many additional bioinformatically predicted prophages were not spontaneously active, with IS transposases strongly correlated with inactivity. Previous studies have shown domesticated prophages are widespread [2, 68], with our results suggesting IS insertion may be an important cause of prophage unviability. This expands upon indications of IS-mediated prophage domestication in a study of eight *E. coli* genomes where IS elements were found in predicted prophage regions [69], with several other examples also known [70, 71].

Building on existing read-mapping tools [32, 33], VIP-Seq’s combination of high sensitivity and quantification of all simultaneously induced prophages sets it apart. We also compared VIP-Seq quantification to EPI and PFU counts. When quantifying a highly efficient phage-host pair (such as T4 and *E. coli* MG1655), PFU counts will provide a very accurate titre. When efficient pairings are not available however, untargeted approaches provide better estimates. Although VIP-Seq moderately underestimated titres compared to EPI in all samples due to DNA loss, EPI will also count other fluorescent particles present in a spent bacterial supernatant [62]. This becomes significant when the SIP titre is low and may explain the high EPI count of the W02_4 supernatant, where both other methods measured no SIPs.

However, this type of read-mapping approach may struggle to identify precise boundaries of transducible phages, PBSX-like gene transfer agents, and other forms of encapsulated gene transfer [33]. This is perhaps why precise boundaries were not found for two spontaneously induced prophages and two mitomycin C-induced prophages. Furthermore, we almost certainly did not detect all viable prophages; even with a sensitive detection limit, there is no single induction trigger for all prophages. Using mitomycin C we discovered two additional inducible prophages, but this was not an exhaustive search, and the number of potential induction triggers is immense. Read-mapping approaches like VIP-Seq are powerful and versatile tools for investigating prophage activity and will aid further investigation into the dynamics of prophages.

The impact of prophage activity in agriculture is potentially great. Although temperate phages are generally considered unsuited for biocontrol due to their tendency to integrate as prophages [10], we find many phyllosphere prophages are already engaged in bacterial warfare. These results also underscore the importance of prophages in designing beneficial microbial SynComs [21]. Although seldom considered in this context, many SynCom members are likely to contain active prophages. Some of these may kill pathogenic strains and promote the establishment of beneficial communities, while others may eliminate desirable plant-beneficial bacteria or spread harmful genes. Regardless, they should not be ignored. Linking traditional phage-based biocontrol with traditional bacteria-based SynComs, prophages represent a potential toolbox for the next generation of plant microbiome engineering and sustainable agriculture.

### Supplementary information

Supplementary Table S1

Supplementary Table S2

Supplementary Figure S3

Supplementary Table S4

Supplementary Table S5

Supplementary Table S6

Supplementary Table S7

Supplementary Table S8

Supplementary Table S9

## Data Availability

The sequencing data used and described in this study has been uploaded to NCBI under the Bioproject accession number PRJNA951732. For any inquiries, please contact PED (ped@plen.ku.dk).

## References

[1] Clokie MR, Millard AD, Letarov AV, Heaphy S (2011). Phages in nature. Bacteriophage.

[2] Ramisetty BCM, Sudhakari PA (2019). Bacterial ‘grounded’ prophages: hotspots for genetic renovation and innovation. Front Genet.

[3] Lopez-Leal G, Camelo-Valera LC, Hurtado-Ramirez JM, Verleyen J, Castillo-Ramirez S, Reyes-Munoz A (2022). Mining of thousands of prokaryotic genomes reveals high abundance of prophages with a strictly narrow host range. mSystems.

[4] Roach M, McNair K, Michalczyk M, Giles S, Inglis L, Pargin E (2022). Philympics 2021: prophage predictions perplex programs. F1000Research.

[5] Gama JA, Reis AM, Domingues I, Mendes-Soares H, Matos AM, Dionisio F (2013). Temperate bacterial viruses as double-edged swords in bacterial warfare. PLoS One.

[6] Pleska M, Lang M, Refardt D, Levin BR, Guet CC (2018). Phage-host population dynamics promotes prophage acquisition in bacteria with innate immunity. Nat Ecol Evol.

[7] Howard-Varona C, Hargreaves KR, Abedon ST, Sullivan MB (2017). Lysogeny in nature: mechanisms, impact and ecology of temperate phages. ISME J.

[8] Bondy-Denomy J, Qian J, Westra ER, Buckling A, Guttman DS, Davidson AR (2016). Prophages mediate defense against phage infection through diverse mechanisms. ISME J.

[9] Li XY, Lachnit T, Fraune S, Bosch TCG, Traulsen A, Sieber M (2017). Temperate phages as self-replicating weapons in bacterial competition. J R Soc Interface.

[10] Frampton RA, Pitman AR, Fineran PC (2012). Advances in bacteriophage-mediated control of plant pathogens. Int J Microbiol.

[11] Raya RR, H’Bert EM (2009). Isolation of Phage via Induction of Lysogens. Methods Mol Biol.

[12] Silpe JE, Wong JWH, Owen SV, Baym M, Balskus EP (2022). The bacterial toxin colibactin triggers prophage induction. Nature.

[13] Bruce JB, Lion S, Buckling A, Westra ER, Gandon S (2021). Regulation of prophage induction and lysogenization by phage communication systems. Curr Biol.

[14] Nanda AM, Heyer A, Kramer C, Grunberger A, Kohlheyer D, Frunzke J (2014). Analysis of SOS-induced spontaneous prophage induction in *Corynebacterium glutamicum* at the Single-Cell Level. J Bacteriol.

[15] Nanda AM, Thormann K, Frunzke J (2015). Impact of spontaneous prophage induction on the fitness of bacterial populations and host-microbe interactions. J Bacteriol.

[16] Owen SV, Wenner N, Canals R, Makumi A, Hammarlof DL, Gordon MA (2017). characterization of the prophage repertoire of African *Salmonella Typhimurium* ST313 reveals high levels of spontaneous induction of novel phage BTP1. Front Microbiol.

[17] Czyz A, Los M, Wrobel B, Wegrzyn G (2001). Inhibition of spontaneous induction of lambdoid prophages in *Escherichia coli* cultures: simple procedures with possible biotechnological applications. BMC Biotechnol.

[18] Tan DM, Hansen MF, de Carvalho LN, Roder HL, Burmolle M, Middelboe M (2020). High cell densities favor lysogeny: induction of an H20 prophage is repressed by quorum sensing and enhances biofilm formation in *Vibrio anguillarum*. ISME J.

[19] Henrot C, Petit MA (2022). Signals triggering prophage induction in the gut microbiota. Mol Microbiol.

[20] Lugli GA, Milani C, Turroni F, Tremblay D, Ferrario C, Mancabelli L (2016). Prophages of the genus *Bifidobacterium* as modulating agents of the infant gut microbiota. Environ Microbiol.

[21] de Souza RSC, Armanhi JSL, Arruda P (2020). From microbiome to traits: designing synthetic microbial communities for improved crop resiliency. Front Plant Sci.

[22] Morella NM, Gomez AL, Wang G, Leung MS, Koskella B (2018). The impact of bacteriophages on phyllosphere bacterial abundance and composition. Mol Ecol.

[23] Forero-Junco LM, Alanin KWS, Djurhuus AM, Kot W, Gobbi A, Hansen LH (2022). Bacteriophages roam the wheat phyllosphere. Viruses.

[24] Degnan PH, Moran NA (2008). Evolutionary genetics of a defensive facultative symbiont of insects: exchange of toxin-encoding bacteriophage. Mol Ecol.

[25] Arndt D, Grant JR, Marcu A, Sajed T, Pon A, Liang YJ (2016). PHASTER: a better, faster version of the PHAST phage search tool. Nucleic Acids Res.

[26] Kieft K, Zhou ZC, Anantharaman K (2020). VIBRANT: automated recovery, annotation and curation of microbial viruses, and evaluation of viral community function from genomic sequences. Microbiome.

[27] Alexeeva S, Martinez JAG, Spus M, Smid EJ (2018). Spontaneously induced prophages are abundant in a naturally evolved bacterial starter culture and deliver competitive advantage to the host. BMC Microbiol.

[28] Zaburlin D, Mercanti DJ, Quiberoni A (2017). A fast PCR-based method for the characterization of prophage profiles in strains of the *Lactobacillus casei* group. J Virol Methods.

[29] Lorenz N, Reiger M, Toro-Nahuelpan M, Brachmann A, Poettinger L, Plener L (2016). Identification and initial characterization of prophages in *Vibrio campbellii*. PLoS One.

[30] Kieft K, Anantharaman K (2022). Deciphering active prophages from metagenomes. mSystems.

[31] Turkington CJR, Abadi NN, Edwards RA, Grasis JA hafeZ: Active prophage identification through read mapping. bioRxiv. 2021:2021.07.21.

[32] Tang KH, Wang WQ, Sun YM, Zhou YQ, Wang PX, Guo YX (2021). Prophage tracer: precisely tracing prophages in prokaryotic genomes using overlapping split-read alignment. Nucleic Acids Res.

[33] Kleiner M, Bushnell B, Sanderson KE, Hooper LV, Duerkop BA (2020). Transductomics: sequencing-based detection and analysis of transduced DNA in pure cultures and microbial communities. Microbiome.

[34] Kolmogorov M, Yuan J, Lin Y, Pevzner PA (2019). Assembly of long, error-prone reads using repeat graphs. Nat Biotechnol.

[35] Ltd. Oxford Nanopore Technologies. Medaka (v1.5.0).

[36] Chaumeil PA, Mussig AJ, Hugenholtz P, Parks DH (2022). GTDB-Tk v2: memory friendly classification with the genome taxonomy database. Bioinformatics.

[37] Jain C, Rodriguez-R LM, Phillippy AM, Konstantinidis KT, Aluru S (2018). High throughput ANI analysis of 90K prokaryotic genomes reveals clear species boundaries. Nat Commun.

[38] Seemann T (2014). Prokka: rapid prokaryotic genome annotation. Bioinformatics.

[39] Steinegger M, Soding J (2017). MMseqs2 enables sensitive protein sequence searching for the analysis of massive data sets. Nat Biotechnol.

[40] Tesson F, Herve A, Mordret E, Touchon M, d’Humieres C, Cury J (2022). Systematic and quantitative view of the antiviral arsenal of prokaryotes. Nat Commun.

[41] Nobrega FL, Walinga H, Dutilh BE, Brouns SJJ (2020). Prophages are associated with extensive CRISPR-Cas auto-immunity. Nucleic Acids Res.

[42] Fu LM, Niu BF, Zhu ZW, Wu ST, Li WZ (2012). CD-HIT: accelerated for clustering the next-generation sequencing data. Bioinformatics.

[43] Krueger F. Babraham Bioinformatics. github.com/FelixKreuger/TrimGalore v0.6.6. 2021.

[44] Bankevich A, Nurk S, Antipov D, Gurevich AA, Dvorkin M, Kulikov AS (2012). SPAdes: a new genome assembly algorithm and its applications to single-cell sequencing. J Comput Biol.

[45] Kot W, Vogensen FK, Sorensen SJ, Hansen LH (2014). DPS - a rapid method for genome sequencing of DNA-containing bacteriophages directly from a single plaque. J Virol Methods.

[46] Moraru C, Varsani A, Kropinski AM (2020). VIRIDIC-A novel tool to calculate the intergenomic similarities of prokaryote-infecting viruses. Viruses.

[47] González-Tortuero EST, Velayudhan V, Shkoporov AN, Draper LA, Stockdale SR, Ross RP, et al. VIGA: a sensitive, precise and automatic de novo VIral Genome Annotator. bioRxiv. 2018:07.03.18.

[48] Steinegger M, Meier M, Mirdita M, Vohringer H, Haunsberger SJ, Soding J (2019). HH-suite3 for fast remote homology detection and deep protein annotation. BMC Bioinforma.

[49] Gilchrist CLM, Chooi YH (2021). clinker & clustermap.js: automatic generation of gene cluster comparison figures. Bioinformatics.

[50] R Core Team. R: A language and environment for statistical computing. Vienna, Austria: R Foundation for Statistical Computing; 2023.

[51] Wickham H ggplot2: Elegant Graphics for Data Analysis. 2016.

[52] McMurdie PJ, Holmes S (2013). phyloseq: an R package for reproducible interactive analysis and graphics of microbiome census data. PLoS One.

[53] Guangchuang Y, Smith D, Zhu H, Guan Y, Tsan-Yuk Lam T (2017). ggtree: an R package for visualization and annotation of phylogenetic trees with their covariates and other associated data. Methods Ecol Evol.

[54] Xu S, Dai Z, Guo P, Fu X, Liu S, Zhou L (2021). ggtreeExtra: compact visualization of richly annotated phylogenetic data. Mol Biol Evol.

[55] Wilke CO ggridges: Ridgeline Plots in ‘ggplot2’. 2022.

[56] Sjoberg D ggsankey. 2021.

[57] de Sousa JAM, Rocha EPC (2022). To catch a hijacker: abundance, evolution and genetic diversity of P4-like bacteriophage satellites. Philos T R Soc B.

[58] Chen J, Quiles-Puchalt N, Chiang YN, Bacigalupe R, Fillol-Salom A, Chee MSJ (2018). Genome hypermobility by lateral transduction. Science.

[59] Fillol-Salom A, Bacigalupe R, Humphrey S, Chiang YN, Chen J, Penades JR (2021). Lateral transduction is inherent to the life cycle of the archetypical *Salmonella* phage P22. Nat Commun.

[60] Humphrey S, Fillol-Salom A, Quiles-Puchalt N, Ibarra-Chavez R, Haag AF, Chen J (2021). Bacterial chromosomal mobility via lateral transduction exceeds that of classical mobile genetic elements. Nat Commun.

[61] Breitbart M, Wegley L, Leeds S, Schoenfeld T, Rohwer F (2004). Phage community dynamics in hot springs. Appl Environ Micro.

[62] Surre J, Saint-Ruf C, Collin V, Orenga S, Ramjeet M, Matic I (2018). Strong increase in the autofluorescence of cells signals struggle for survival. Sci Rep..

[63] Shao Y, Wang IN (2009). Effect of late promoter activity on bacteriophage lambda fitness. Genetics.

[64] Siguier P, Perochon J, Lestrade L, Mahillon J, Chandler M (2006). ISfinder: the reference centre for bacterial insertion sequences. Nucleic Acids Res.

[65] Toussaint A, Rice PA (2017). Transposable phages, DNA reorganization and transfer. Curr Opin Microbiol.

[66] Zhang M, Hao Y, Yi Y, Liu S, Sun Q, Tan X (2023). Unexplored diversity and ecological functions of transposable phages. ISME J.

[67] Kidambi SP, Ripp S, Miller RV (1994). Evidence for phage-mediated gene-transfer among *Pseudomonas aeruginosa* Strains on the Phylloplane. Appl Environ Micro.

[68] Bobay LM, Touchon M, Rocha EPC (2014). Pervasive domestication of defective prophages by bacteria. Proc Natl Acad Sci USA.

[69] Ooka T, Ogura Y, Asadulghani M, Ohnishi M, Nakayama K, Terajima J (2009). Inference of the impact of insertion sequence (IS) elements on bacterial genome diversification through analysis of small-size structural polymorphisms in *Escherichia coli* O157 genomes. Genome Res.

[70] Cai H, Zhu Y, Hu D, Li Y, Leptihn S, Loh B (2021). Co-harboring of Novel bla (KPC-2) plasmid and integrative and conjugative element carrying Tn6203 in Multidrug-Resistant *Pseudomonas aeruginosa*. Front Microbiol.

[71] McDonough MA, Butterton JR (1999). Spontaneous tandem amplification and deletion of the shiga toxin operon in *Shigella dysenteriae* 1. Mol Microbiol.

