# Supplementary figures and images for: Widespread and largely unknown prophage activity, diversity, and function in two genera of wheat phyllosphere bacteria
Source: ISME J. 2023 Nov 2;17(12):2415–25. doi: 10.1038/s41396-023-01547-1 (PMC10689766; doi:10.1038/s41396-023-01547-1)

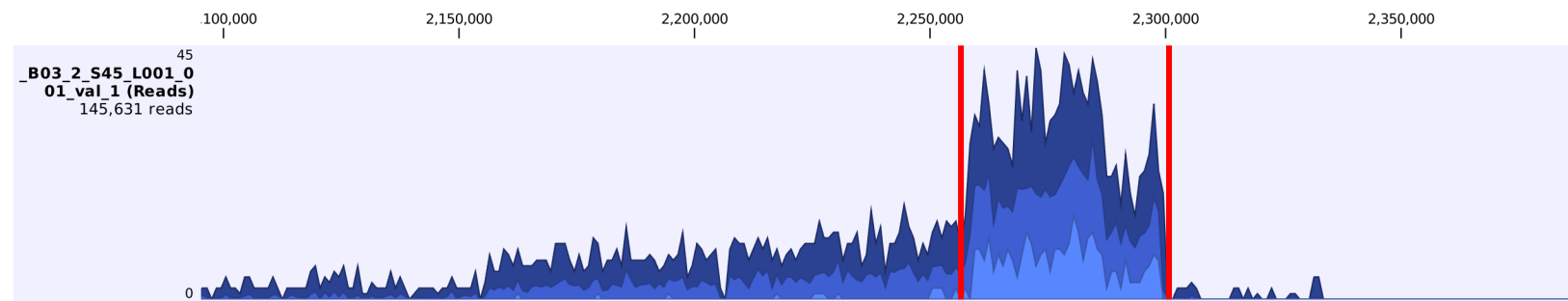

**A**

**Storsmeden\_A**

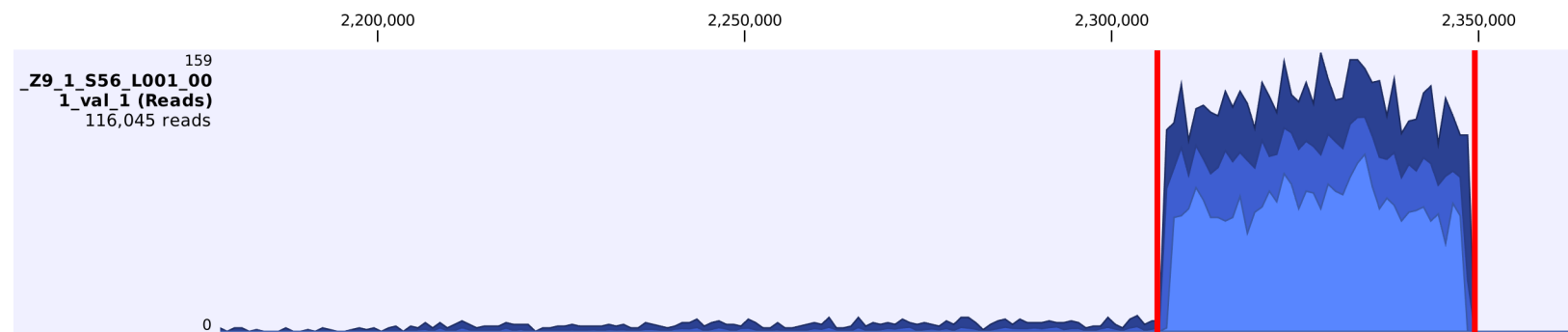

**B**

**Trolltunga\_A**

Supplement: Supplementary file 3 — Supplementary Figure S3 [file 41396_2023_1547_MOESM3_ESM.pdf]
